# Relationship between CMR-derived parameters of ischemia/reperfusion injury and the timing of CMR after reperfused ST-segment elevation myocardial infarction

**DOI:** 10.1186/s12968-018-0474-7

**Published:** 2018-07-23

**Authors:** Pier-Giorgio Masci, Anna Giulia Pavon, Olivier Muller, Juan-Fernando Iglesias, Gabriella Vincenti, Pierre Monney, Brahim Harbaoui, Eric Eeckhout, Juerg Schwitter

**Affiliations:** 1 Centre of Cardiac Magnetic Resonance, University Hospital Lausanne-CHUV, Lausanne, Switzerland; 2 Cardiology Division, Heart & Vessels Department, Lausanne University Hospital-CHUV, BH-09-792 Rue de Bugnon 46, CH-1011 Lausanne, Vaud Switzerland; 3 Cardio-Thoracic-Vascular Department, San Raffaele’s Scientific Institute, Milan, Italy

**Keywords:** Myocardial infarction, Cardiovascular magnetic resonance, T2-mapping, T1-mapping, Extracellular volume, Myocardial edema

## Abstract

**Background:**

To investigate the influence of cardiovascular magnetic resonance (CMR) timing after reperfusion on CMR-derived parameters of ischemia/reperfusion (I/R) injury in patients with ST-segment elevation myocardial infarction (STEMI).

**Methods:**

The study included 163 reperfused STEMI patients undergoing CMR during the index hospitalization. Patients were divided according to the time between revascularization and CMR (T_revasc-CMR_: Tertile-1 ≤ 43; 43 < Tertile-2 ≤ 93; Tertile-3 > 93 h). T2-mapping derived area-at-risk (AAR) and intramyocardial-hemorrhage (IMH), and late gadolinium enhancement (LGE)-derived infarct size (IS) and microvascular obstruction (MVO) were quantified. T1-mapping was performed before and > 15 min after Gd-based contrast-agent administration yielding extracellular volume (ECV) of infarct.

**Results:**

Main factors influencing I/R injury were homogenously balanced across T_revasc-CMR_ tertiles. T2 values of infarct and remote regions increased with increasing T_revasc-CMR_ tertiles (infarct: 60.0 ± 4.9 vs 63.5 ± 5.6 vs 64.8 ± 7.5 ms; *P* < 0.001; remote: 44.3 ± 2.8 vs 46.1 ± 2.8 vs ± 46.1 ± 3.0; *P* = 0.001). However, T2 value of infarct largely and significantly exceeded that of remote myocardium in each tertile yielding comparable T2-mapping-derived AAR extent throughout T_revasc-CMR_ tertiles (17 ± 9% vs 19 ± 9% vs 18 ± 8% of LV, respectively, *P* = 0.385). Similarly, T2-mapping-based IMH detection and quantification were independent of T_revasc-CMR_. LGE-derived IS and MVO were not influenced by T_revasc-CMR_ (IS: 12 ± 9% vs 12 ± 9% vs 14 ± 9% of LV, respectively, *P* = 0.646). In 68 patients without MVO, T1-mapping based ECV of infarct region was comparable across T_revasc-CMR_ tertiles (*P* = 0.470).

**Conclusion:**

In STEMI patients, T2 values of infarct and remote myocardium increase with increasing CMR time after revascularization. However, these changes do not give rise to substantial variation of T2-mapping-derived AAR size nor of other CMR-based parameters of I/R.

**Trial registration:**

ISRCTN03522116. Registered 30.4.2018 (retrospectively registered).

## Background

Cardioprotection after primary percutaneous coronary intervention (PPCI) involves therapy which reduced myocardial damage due to ischemia/reperfusion (I/R), with the aim of minimizing infarct size in patients with ST-segment elevation myocardial infarction (STEMI) [1]. This field is rapidly expanding and relies heavily on non-invasive imaging-based quantitative metrics of ischemic myocardial damage. In this sense, cardiovascular magnetic resonance (CMR) plays a crucial role given its ability to non-invasively depict and quantify the diverse components of ischemic damage with high precision and reproducibility [2]. Accordingly, several CMR-based parameters of I/R have been adopted as surrogate end-points in several cardioprotective studies, obviating costly and ethically questionable large clinical trials [2, 3]. Early after STEMI, CMR allows to measure IS, infarct-related myocardial edema, an estimate of area-at risk (AAR), as well as intramyocardial hemorrhage (IMH) and microvascular obstruction (MVO), two markers of microvasculature damage [2, 4]. However, recent studies have cast doubts about the reliability of these parameters when CMR is carried out early after reperfusion [5–12]. In particular, they underpinned that infarct-related myocardial edema features a highly dynamic course after reperfusion rising concerns about the optimal timing of CMR. However, these studies were limited by small and selective study populations, and the time course of edema was rather heterogeneous as compared to pivotal experimental studies conducted in well-controlled I/R animal models [6–9]. Indeed, in STEMI patients the pattern of infarct-related edema may be influenced by disparate poorly quantifiable factors, including the severity and duration of ischemia as well as spontaneous cardioprotection phenomena [9, 13]. Most important, the above studies relied largely on T2-weighted CMR for AAR quantification, which is less accurate, precise and robust than novel mapping techniques for edema quantification [14, 15].

Based on these premises, we studied a cohort of consecutive unselected reperfused STEMI patients using comprehensive CMR including mapping techniques during the index hospitalization to assess whether and to which extent the dynamics of edema influence CMR-derived parameters of I/R damage.

## Methods

### Study population

This is a pre-specified study originating from a single-center registry which prospectively included STEMI patients undergoing CMR during the index hospitalization. Between July 2014 and June 2017, 195 patients with the diagnosis of STEMI were assessed for study inclusion at Lausanne University Hospital. Patients were included if they were older than 18 years, met the electrocardiogram (ECG) criteria for STEMI and were treated by PPCI. Exclusion criteria included refractory cardiogenic shock, prior myocardial infarction or coronary revascularization, claustrophobia, and glomerular filtration rate < 30 mL/min/1.73m^2^. The study was approved by institutional review board and all patients provided written informed consent.

### Cardiovascular magnetic resonance protocol

All patients underwent CMR at 1.5 Tesla (Siemens Healthineers, Aera-Magneton, Erlangen-Germany). All studies were performed using dedicated cardiac software, 32-channel phased-array surface receiver coil and ECG triggering. Cine images were acquired using a breath-hold balanced steady-state free-precession (bSSFP) in long-axis and short-axis views. A stack of short-axis slices was acquired to quantify left ventricular (LV) volumes, mass and ejection-fraction. T2-mapping data were acquired by breath-hold T2-prepared bSSFP in the same orientation of short-axis cine images [14]. T2-maps were readily evaluated by an experienced operator during CMR scan, and a single short-axis target-slice was selected in the middle of infarction (*Supplemental-Material*). Native and post-contrast T1-mapping data were acquired in the target-slice using ECG-triggered breath-hold MOdified Look-Locker Inversion Recovery (MOLLI) sequence with a 5(3)3 and 4(1)3(1)2 sampling scheme for native and post-contrast T1-maps, respectively [16]. Ten minutes after 0.2 mmol/Kg i.v. bolus of Gadobutrol (Gadovist, Bayer Healthcare, Berlin, Germany), late gadolinium enhancement (LGE) images were acquired using a 2D breath-hold phase-sensitive segmented inversion-recovery gradient echo in the same orientations of cine images. Inversion-time was individually optimized to null normal myocardium. In the target-slice, post-contrast T1-mapping was acquired at the end of LGE imaging (> 15 min after Gadobutrol bolus injection). Sequences and protocol are detailed in the *Supplemental-Material*.

### Image analysis

All studies were analyzed by a single operator with > 10 years experience in CMR (PGM) using GTVolume software (GyroTools, Version 2.2.1, Zurich-Switzerland). LV volumes, mass, and ejection-fraction were calculated by manually delineating endocardial and epicardial borders in the stack of short-axis cine images. For each T2-map, endocardial and epicardial borders were manually traced. A region-of-interest was drawn in the central-layer of the remote myocardium, and edema was quantified as myocardium with T2 value≥2 standard-deviation of remote myocardium (≥upper limit of the 95% confidence-intervals of the mean T2 values of remote myocardium) [17]. IMH was defined as myocardium within edema showing T2 value< 2 standard-deviation of mean T2 of the remote myocardium. Automatically-defined contours of AAR were manually corrected by adding or removing pixels which were incorrectly disregarded or excluded by the software, respectively. AAR and IMH were quantified by summing edema and IMH measured in each slice, and expressed as absolute (grams) or % of LV mass (% of LV). In hemorrhagic infarcts, IMH was added to edema for AAR quantification. Mean T2 values of AAR, IMH, and remote myocardium were measured. Native and post-contrast T1 values of the target-slice were measured in the infarct and remote myocardium. Extracellular volume (ECV) in the infarcted and remote regions was calculated by adopting the validated equation (*Supplemental Material*). On LGE images, infarct size was quantified as myocardium with a signal intensity exceeding the mean signal intensity of remote myocardium by >5SD [18]. MVO was defined as hypointense core embedded in the hyperenhanced myocardium and, if present, was included in hyper-enhanced myocardium for infarct size quantification. Infarct size and MVO were expressed as absolute value (gram) or as percentage of LV mass (% of LV). Myocardial salvage index is calculated as: [(AAR − IS)/AAR]x100.

### Determination of the interval between revascularization and CMR

The time (day and hour) of PPCI and CMR were recorded. First stent deployment in the infarcted-related artery was considered as the time of reperfusion, whereas beginning of CMR exam during the index hospitalization was used as CMR time. Time between reperfusion and CMR was calculated (T_Revasc-CMR_).

### Statistical analysis

Continuous data were expressed as mean ± SD or as median (25th–75th percentile), and categorical data as frequency with percentage (%). Comparison of categorical variables between groups was performed by χ2 test, or by Fisher exact test if the expected cell count was < 5. One-way ANOVA or nonparametric Kruskal-Wallis tests were used as appropriate to compare continuous variables between T_revasc-CMR_ tertiles. Bonferroni post-hoc was used to test differences between two independent groups. All tests were 2 tailed, and *P* < 0.05 was considered statistically significant. Analyses were performed with SSPS version 21 (International Business Machines, Inc., Armonk, New York, USA).

## Results

### Study population

**Fig. 1:**
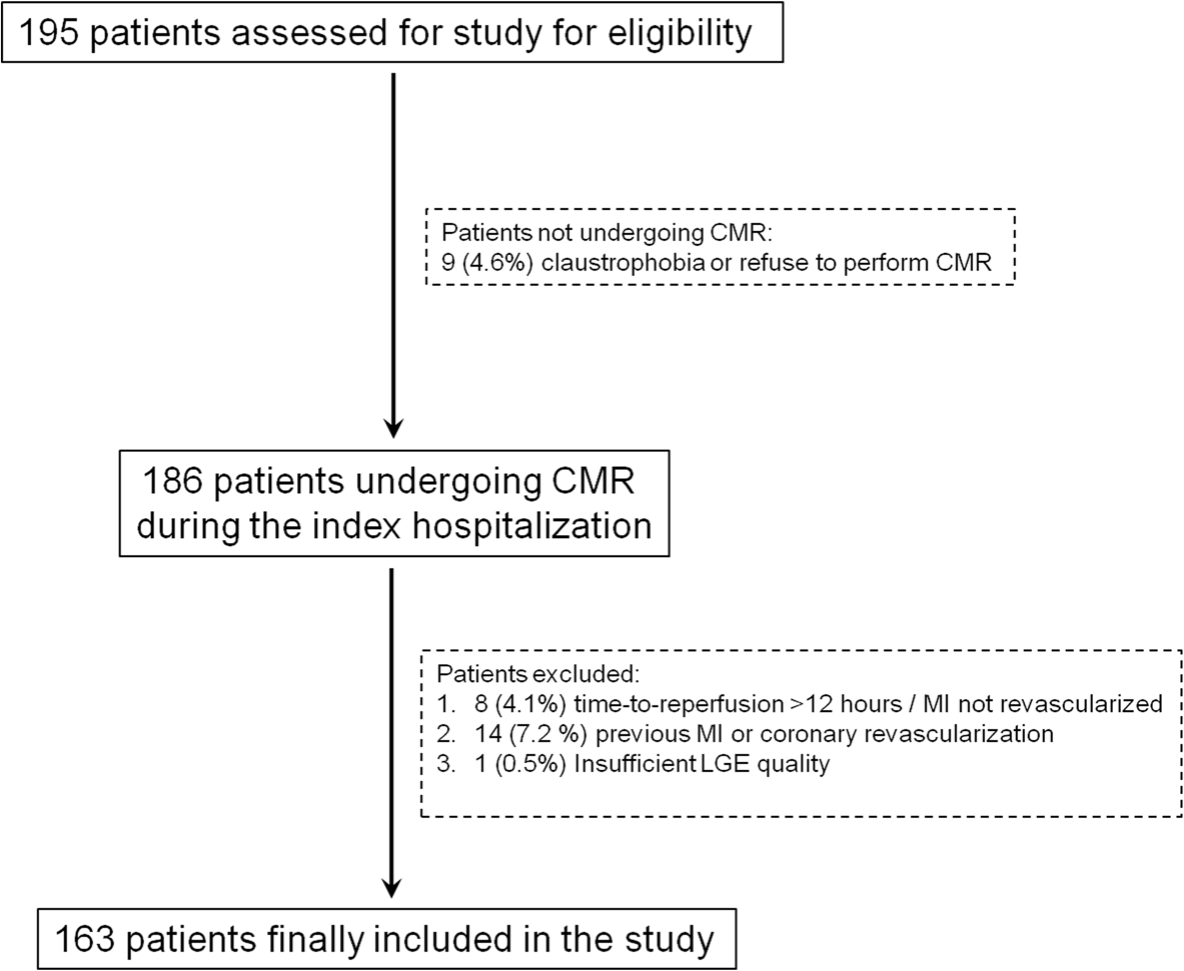
Study Protocol

Among the 195 patients evaluated for study eligibility, 9 patients did not undergo CMR because of claustrophobia or refusal of the exam. Twenty-three patients were excluded after CMR because of time-to-reperfusion exceeded 12 h from symptoms onset, previous myocardial infarction / coronary revascularitation or insufficient LGE quality. One-hundred-sixty-three patients were finally included (Fig. 1). Patients were then divided according to T_revasc-CMR_ tertiles (Table 1). Fifty-three (32%) patients showed pre-PPCI blood flow in the ischemic myocardium at risk via the infarct-related artery (TIMI-flow-grade ≥ 2; *n* = 42) or collaterals (Rentrop-grade ≥ 2; *n* = 11). Patients in Tertile-3 tended to be older presenting with lower systolic blood pressure at hospital admission than patients in Tertile-1 or Tertile-2. Except for these variables, baseline characteristics were comparables across T_Revasc-CMR_ tertiles (Table 2).

**Table 1:** Tertiles of the time elapsed from revascularization to CMR exam

Tertile	Number of patients	Time (hour)	Median (25-75th percentiles), hours	Median (25-75th percentiles), days	Min / Max (hours)
Tertile-1	54	≤43.2	23.4 (20.3–33.5)	1.0 (0.8–1.4)	6.2 / 42.5
Tertile-2	55	> 43.2 but ≤93.1	65.7 (50.1–79.1)	2.7 (2.1–3.3)	43.5 / 93.0
Tertile-3	54	> 93.1	127.1 (108.9–187.8)	5.3 (4.5–7.8)	93.3 / 332.4

**Table 2:** Baseline Characteristics

Characteristics	Overall (*n* = 163)	Tertile-1 (*n* = 54)	Tertile-2 (*n* = 55)	Tertile-3 (*n* = 54)	*P*-value
Age (years)	60 ± 13	60 ± 14	57 ± 14	63 ± 11	0.054
Male Gender * n*, (%)	120 (74)	40 (74)	39 (71)	41 (76)	0.835
FH for CAD *n*, (%)	19 (12)	9 (17)	6 (11)	4 (7)	0.318
Diabetes *n*, (%)	24 (15)	7 (13)	5 (9)	12 (22)	0.139
Hypertension *n* (%)	90 (55)	27 (50)	28 (51)	35 (65)	0.219
Hypercholesterolemia *n*, (%)	92 (53)	30 (56)	33 (60)	29 (54)	0.658
Active Smoking *n*, (%)	66 (40)	20 (37)	26 (47)	20 (37)	0.453
Prodromal Angina *n*. (%)	55 (34)	19 (35)	20 (36)	16 (30)	0.731
Time-to-PPCI (min)	195 (128–270)	209 (129–270)	173 (125–208)	197 (149–303)	0.111
Systolic blood pressure (mmHg)	119 ± 21	124 ± 22	120 ± 21	114 ± 20	0.061
Diastolic blood pressure (mmHg)	70 ± 14	71 ± 15	72 ± 14	68 ± 13	0.368
Heart Rate (bpm)	75 ± 16	74 ± 14	73 ± 15	78 ± 19	0.165
RPP before PPCI (mmHg∙bpm)	9032 ± 2713	9296 ± 2780	8779 ± 2547	9027 ± 2831	0.613
Infarct related artery *n*, (%)					0.537
LAD	74 (45)	28 (52)	23 (42)	23 (43)	
RCA	62 (38)	17 (31)	25 (46)	20 (37)	
LCX	27 (17)	9 (17)	7 (13)	11 (20)	
Anterior Infarction *n*, (%)	74 (45)	28 (52)	23 (41)	23 (43)	0.506
Non-IRA critical stenosis *n*, (%)					0.865
0	79 (49)	28 (52)	28 (51)	23 (43)	
1	51 (31)	15 (25)	17 (31)	19 (35)	
2	33 (20)	11 (20)	10 (18)	12 (22)	
TIMI flow-grade pre-PPCI *n*, (%)					0.237
0,1	121 (74)	40(74)	37 (67)	44 (82)	
2,3	42 (26)	14 (26)	18 (33)	10 (19)	
TIMI flow-grade post-PPCI *n*, (%)					0.444
0,1	2 (1)	0(0)	1 (2)	1 (2)	
2,3	161 (99)	54 (100)	54 (98)	53 (98)	
Rentrop flow-grade *n*, (%)					0.152
0,1	152 (93)	53 (98)	50 (91)	49 (91)	
2,3	11 (7)	1 (2)	5 (9)	5 (9)	
*Medication at discharge*					
ACEi or ARBs *n*, (%)	160 (98)	53 (98)	53 (96)	54 (100)	0.369
Beta-blockers *n*, (%)	126 (77)	39 (72)	44 (80)	43 (80)	0.552
Statins *n*, (%)	154 (95)	51 (94)	54 (98)	53 (98)	0.458
Diuretics *n*, (%)	13 (8)	2 (4)	4 (7)	7 (13)	0.197

*ACEi* angiotensin-converting enzyme inhibitor, *ARB* angiotensin receptor blocker, *CAD* coronary artery disease, *FH* familial history, *IRA* infarct-related artery, *LAD* left anterior descending artery, LCX:left circumflex, *PPCI* primary percutaneous coronary intervention, *RCA* right coronary artery, *RPP* rate-pressure product, *TIMI* Thrombolysis In Myocardial Infarction

### Differences of AAR, infarct size and microvascular damage and LV functional parameters according to the time between revascularization and CMR

**Fig. 2:**
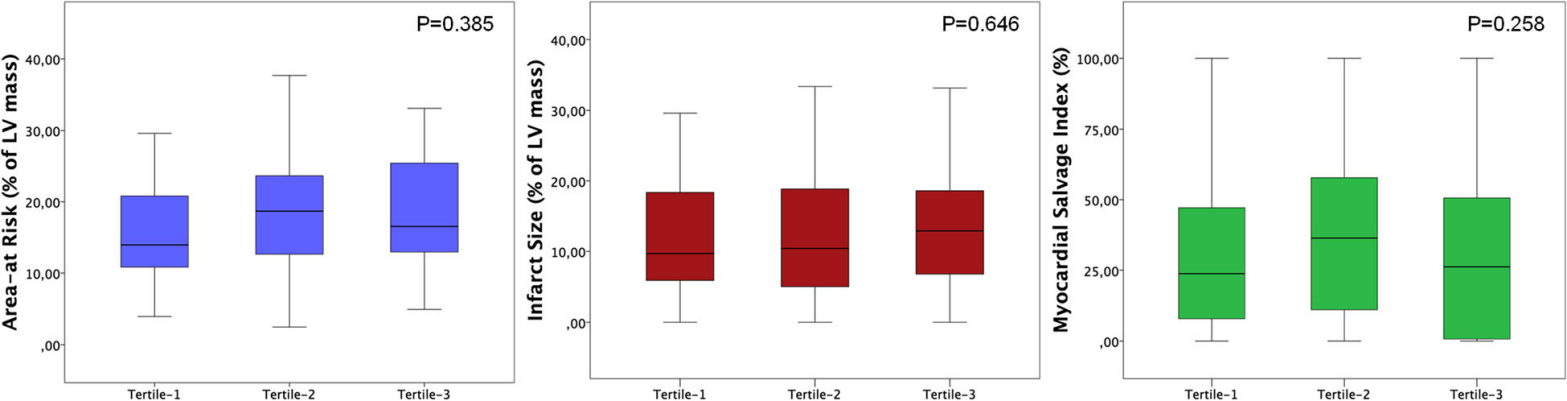
Box-plots of area-at risk, infarct size and myocardial salvage in the tertiles of T_revasc-CMR_

Results are summarized in the Table 2. AAR remained stable across T_Revasc-CMR_ tertiles **(**Fig. 2**)**. Overall 46 (28%) patients showed IMH on T2-mapping. Occurrence and extent of IMH did not differ among T_Revasc-CMR_ tertiles. IS and occurrence and extent of MVO as well as myocardial salvage index were comparable throughout T_Revasc-CMR_ tertiles **(**Fig. 2**)**. LV end-diastolic volume index increased slightly across T_Revasc-CMR_ tertiles, so that patients in Tertile-3 patients had larger volume than those in Tertile-1. LV end-systolic volume index, regional and global systolic function parameters were comparable across T_Revasc-CMR_ tertiles.

### T2 relaxation times in infarct and remote regions

**Fig. 3:**
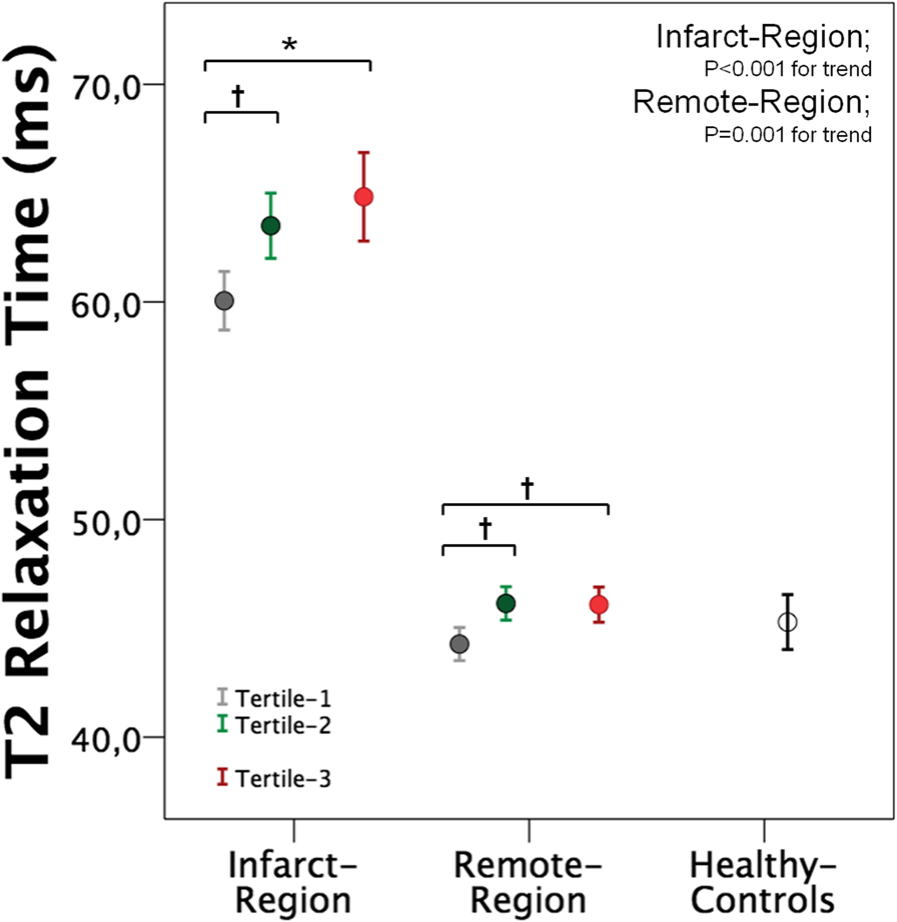
Error Bars showing the mean and 95% confidence intervals of T2 values in infarct and remote regions across tertiles of T_revasc-CMR._ Error bar of healthy controls is reported as reference**.** (Bonferroni’s post-hoc analysis: *P < 0.001; † *P* < 0.05)

**Fig. 4:**
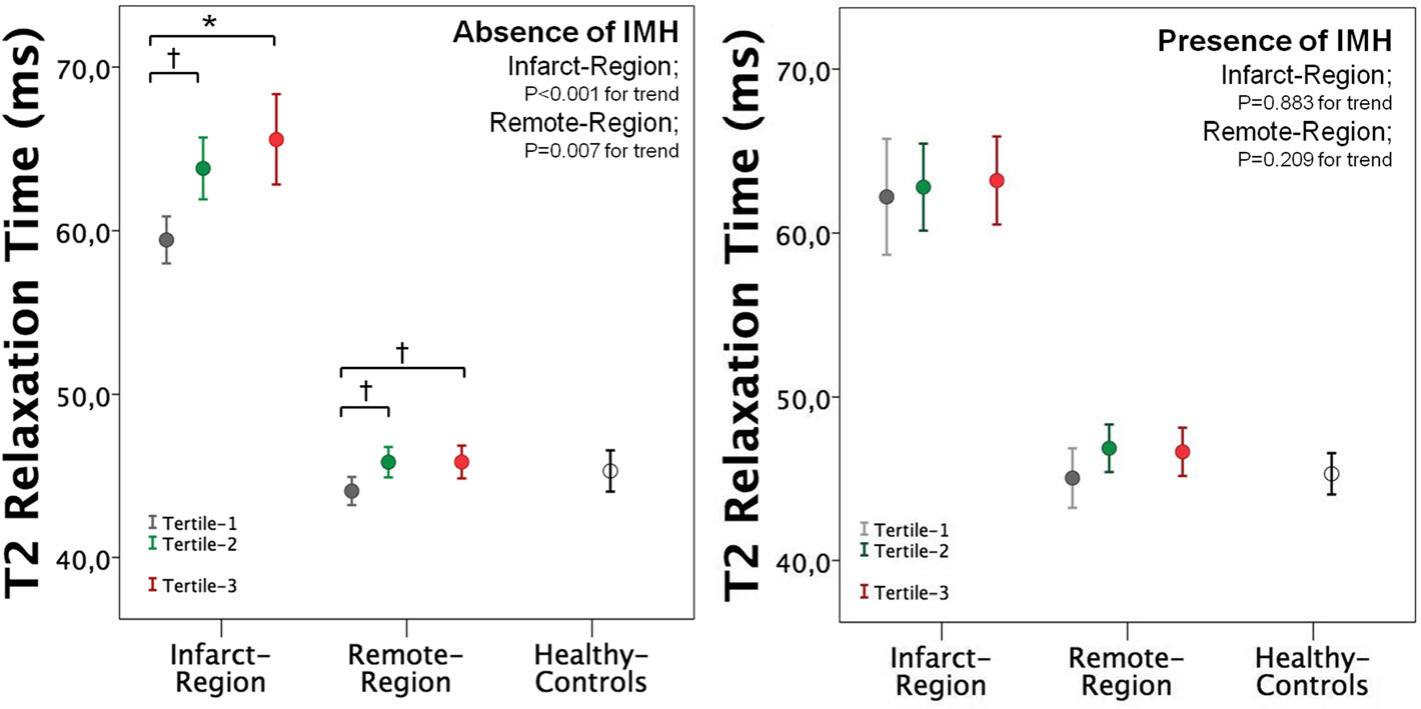
Error Bars showing the mean and 95% confidence intervals of T2 values in infarct and remote regions across tertiles of Trevasc-CMR in patients with and without intramyocardial hemorrage (IMH). Error bar of healthy controls is reported as reference**.** (Bonferroni’s post-hoc analysis: *P < 0.001; † *P* < 0.05)

**Fig. 5:**
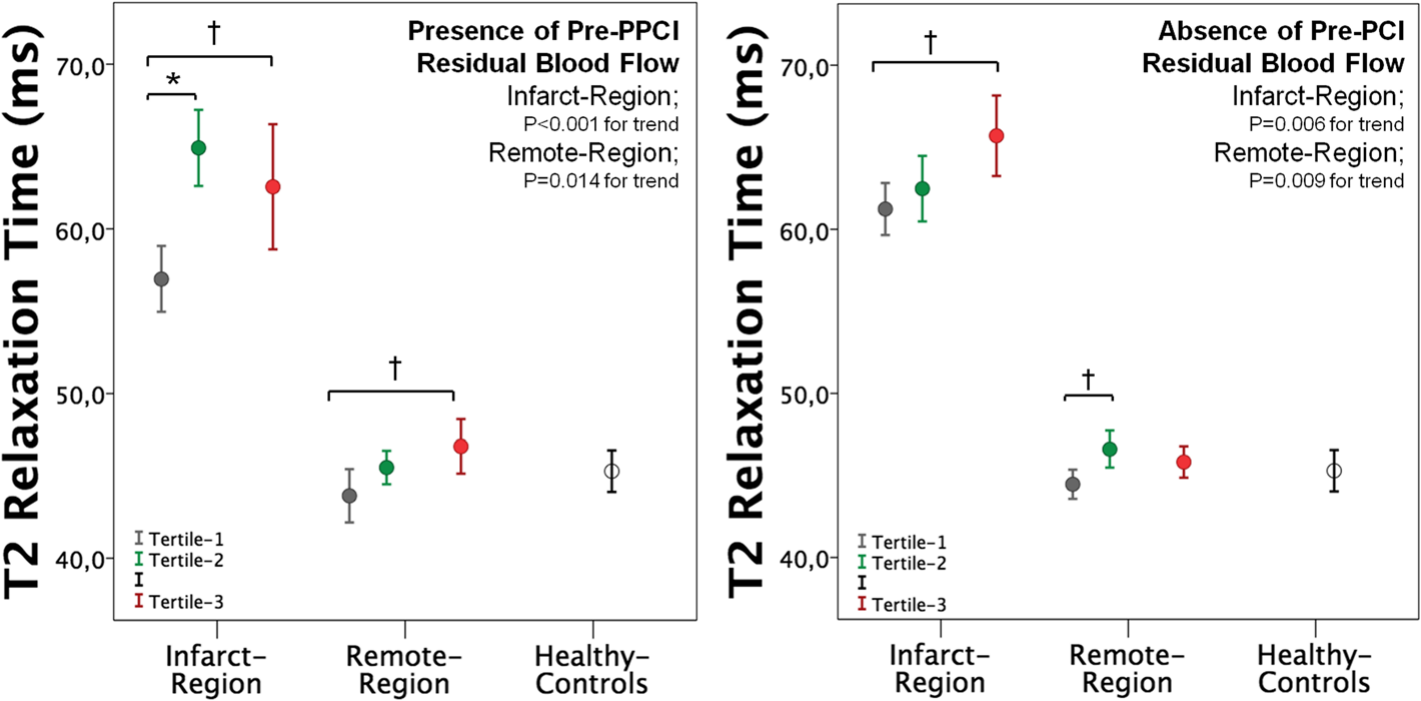
Error bars showing the mean and 95% confidence intervals of T2 values in infarct and remote regions across tertiles of Trevasc-CMR in patients with and without residual pre-PPCI blood flow in the myocardium at risk**. (**Bonferroni’s post-hoc analysis: *P < 0.001; † *P* < 0.05)

Overall T2 of infarct region was consistently higher than that of remote region throughout T_Revasc-CMR_ tertiles, and as compared to normal values of our center (48.5 ± 2.4 ms). Infarct T2 increased between Tertile-1 and Tertile-2 without further increase between Tertile-2 and Tertile-3. A small increase of T2 relaxation time was also observed in the remote myocardium across T_Revasc-CMR_ tertiles (Fig. 3). When patients were divided according to presence or absence of IMH, only patients without IMH showed an increase of infarct T2 values between Tertile-1 and Tertile-2 (Fig. 4). Differently, patients with IMH presented a constant elevation of T2 values within the infarct region throughout T_Revasc-CMR_ tertiles. When comparing T2 values between patients with and without IMH, Tertile-1 patients with IMH tended to have higher infarct T2 value than those without IMH (62.2 ± 5.6 vs 59.4 ± 4.6 ms, *P* = 0.085) (further details in Table 2***;*** *Supplemental Material*). By dichotomizing patients according to presence (*n* = 53, pre-PPCI TIMI flow-grade or Rentrop flow-grade ≥ 2) or absence (*n* = 110; pre-PPCI TIMI flow-grade or Rentrop flow-grades< 2) of residual blood blow in the ischemic myocardium, both subgroups showed a significant increase of T2 values with increasing T_Revasc-CMR_ tertiles in both the infarct and remote regions (Fig. 5). By comparing patients with or without pre-PPCI residual blood flow, Tertile-1 patients with residual blood flow showed lower infarct T2 value than those without (57.0 ± 3.6 vs 61.2 ± 4. ms, *P* = 0.003) (further details in Table 3*; Supplemental Material*).

**Table 3:** CMR Characteristics

Characteristics	Overall (*n* = 163)	Tertile-1 (*n* = 54)	Tertile-2 (*n* = 55)	Tertile-3 (*n* = 54)	*P*-Value For trend
Infarct-related Measures					
AAR (g)	23 ± 12	20 ± 12	23 ± 11	25 ± 13	0.113
AAR (% LV)	18 ± 8	17 ± 9	19 ± 9	18 ± 8	0.385
IMH n, (%)	46 (28)	12 (22)	17 (31)	17 (31)	0.487
IMH extent (g)	2.8 (1.6–5.4)	2.8 (1.5–9.2)	3.0 (2.1–4.8)8	2.4 (1.1–5.4)	0.794
IMH extent (% of LV)	2.0 (1.0–4.1)	2.7 (1.0–7.2)	2.4 (1.3–3.9)	1.9 (0.8–3.9)1	0.681
Infarct size (g)	16 ± 13	15 ± 12	16 ± 12	19 ± 14	0.271
Infarct size (% of LV)	13 ± 9	12 ± 9	12 ± 9	14 ± 9	0.646
MVO n, (%)	73 (45)	23 (43)	25 (46)	25 (46)	0.921
MVO extent (g)	4.0 (2.3–7.9)	3.0 (2.0–4.2)	5.6 (3.0–7.8)	5.0 (2.3–11.4)	0.294
MVO extent (% of LV)	3.8 (1.8–6.9)	2.5 (1.5–4.5)	4.4 (2.0–6.9)	3.8 (1.9–8.0)	0.426
MSI (%)	31 (7–55)	24(8–48)	36 (8–58)	26 (1–51)	0.258
LV functional Parameters					
LV-EDVi (ml/m^2^)	75 ± 19	71 ± 17	75 ± 18	80 ± 21	0.042
LV-ESVi (ml/m^2^)	35 ± 19	33 ± 13	34 ± 18	40 ± 22	0.068
LV-EF (%)	50 ± 12	51 ± 11	52 ± 11	48 ± 15	0.198
LV-Mi (g/m^2^)	66 ± 15	63 ± 15	67 ± 15	69 ± 15	0.129
RelaxationTimes					
*T2-mapping*					
T2 infarct (ms)	62.8 ± 6.4	60.0 ± 4.9†•	63.5 ± 5.6•	64.8 ± 7.5†	< 0.001
T2 remote (ms)	45.5 ± 3.0	44.3 ± 2.8•*	46.1 ± 2.8•	46.1 ± 3.0*	0.001
T2 infarct core (ms)	47.0 (44.9–50.0)	47.5 (46.0–49.7)	45.8 (42.6–48.5)	48.0 (45.8–50.6)	0.071
T1-mapping*					
T1 infarct (ms)*	1220 ± 81	1172 ± 63†•	1235 ± 78•	1248 ± 82†	< 0.001
T1 remote (ms)*	1019 ± 59	1004 ± 37	1019 ± 54	1034 ± 76	0.076
ECV infarct (%)**	46.7 (41.5–52.0)	49.0 (41.6–53.1)	45.6 (41.7–52.9)	45.9 (40.1–49.5)	0.470
ECV remote (%)**	24.5 (22.1–27.1)	25.2 (22.0–27.0)	23.9 (22.3–25.7)	24.6 (22.0–28.3)	0.916

* *n* = 124; **n = 68. Bonferroni’s post-hoc analysis: •,* *P* < 0.05; † *P* < 0.001. *AAR* area at risk, *ECV* extracellular volume, *EDV*i end-diastolic volume index, *EF* ejection-fraction, *ESVi* end-systolic volume index, *IMH* intramyocardial hemorrhage, *LV* left ventricular, *Mi* mass index, *MVO* microvascular obstruction, *MSI* myocardial salvage index

### T1 relaxation time and extracellular volume fraction in the infarct and remote regions

T1 values of infarct and remote regions were measured in the target-slice of 124 patients. Overall, T1 of infarct was higher than that of remote region throughout T_Revasc-CMR_ tertiles, and as compared to normal values of our centre (997 ± 27 ms, range: 943–1051 ms), (Fig. 1-*Supplemental Material*). Infarct T1, paralleling the behavior of T2, increased with the increase of T_Revasc-CMR_ tertiles_,_. Post-hoc analysis revealed that T1 values increased from Tertile-1 to Tertile-2 without further increase from Tertile-2 to Tertile-3. By dichotomizing patients based on the presence or absence of IMH, only those without IMH increased infarct T1 value with the increase of T_Revasc-CMR_ tertiles, mirroring the results of T2 (Fig. 2-*Supplemental Material*). When patients were divided according to pre-PPCI residual blood flow, both subgroups showed significant increase of T1 value in the infarct region with the increase of T_Revasc-CMR_ tertiles (Fig. 3-*Supplemental Material*). We calculated ECV of the infarct and remote regions in patients without MVO (*n* = 68), given that state steady of gadolinium-based contrast agent cannot be reached between MVO (LGE-negative) and infarct (LGE-positive). Infarct and remote ECV values were comparable throughout T_Revasc-CMR_ tertiles (Fig. 4-*Supplemental Material*).

## Discussion

In a cohort of consecutive unselected STEMI patients treated by PPCI and undergoing comprehensive CMR during the index hospitalization, we found that T2-maps-based AAR quantification was independent of the timing of CMR after revascularization (T_Revasc-CMR_). Although T2 values of the infarct region increased with increasing time interval between revascularization and CMR, T2 values of the infarct largely exceeded those of the remote myocardium yielding constant AAR despite the increase of T_Revasc-CMR_. These findings were confirmed irrespective of presence or absence of IMH and of pre-PPCI residual flow in the myocardium at risk. Finally, LGE-dependent metrics, including infarct size and MVO occurrence and its extent, were independent of the timing of CMR after revascularization.

Recent reports generated intense discussion on the dynamic changes of CMR-derived measures of ischemic damage in early post-STEMI phase with particular emphasis on myocardial edema. In elegant experimental studies [6, 7, 9], Fernàndez-Jiménez et al. showed that edema features a bimodal pattern after reperfusion. An initial wave of edema appears abruptly and very early (≤3 h) to be attenuated at 24 h and followed by a second edema wave peaking at 4 to 7 days. The same group described this bimodal pattern in a selected cohort of 16 reperfused STEMI patients undergoing serial CMR (8). In patients, the authors found that T2 values within the ischemic region, as measured by T2-mapping, varied consistently throughout the post-reperfusion phase paralleling the experimental results. Accordingly, AAR, as quantified by T2-weighted short-TI (T2w-STIR), changed substantially with the timing of CMR being significantly lower at 24 h than at hyper-acute (≤3 h) or at 4 and 7 days after reperfusion. In line with these findings, we observed that T2 of the infarct increased with the increase of timing of CMR after revascularization, with a steep augmentation between Tertile-1 (median: 1.0 day) and Tertile-2 (median: 2.7 days) without further change between Tertile-2 and Tertile-3 (median: 5.3 days). However differently from the above study, we found that T2-map-derived AAR did not vary with the timing of CMR after PPCI. This discrepancy can be ascribed to the differences in study populations and, most importantly, to the different techniques utilized for AAR quantification. While Fernàndez-Jiménez’s et al. utilized T2w-STIR, we used T2-mapping, which allows more robust, precise and reproducible estimation of infarct-related edema as compared to T2w-STIR [14, 15]. Different from T2w-STIR imaging, T2-mapping consents to measure T2 lengthening of the ischemic tissue due to increased water content, rendering this techniques particular sensitive to subtle changes in T2 relaxation times [14, 17, 19]. Differently, T2-weightening relies on relative signal intensity differences between ischemic and remote myocardium, which are influenced by several factors in addition to edema per se, such as specific T2-weightening parameters, motion-related signal loss in the lateral wall or selection of window/leveling to display images [15]. Accordingly, slight differences in T2 relaxation times between ischemic and remote myocardium, such as those reported in the experimental and clinical studies by Fernàndez-Jiménez et al. [6–9] at 24-h after reperfusion, might have been overlooked by T2w-STIR leading to underestimation of AAR. In our study, T2 values of infarct myocardium exceeded largely those of remote region irrespective of the time interval between revascularization and CMR, the presence or absence of IMH, and pre-PPCI residual blood flow in the myocardium at risk. Accordingly, we were able to quantify AAR on T2-maps using a semi-automatic threshold-based algorithm in the overall population and specific subgroups [17]. Moreover, the pattern of T1 values in infarct and remote myocardium paralleled closely that of T2, corroborating our study results. Experimentally T1 relaxation time increases in parallel with increasing myocardial water content, and prolonged T1 has been reported in myocardium at risk in STEMI patients [20, 21].

In summary, our data confirm the dynamic course of T2 in ischemic and remote myocardium as reported by others [6–9]. However, these changes do not translate into systematic variation of T2-mapping-based AAR quantification, given the high sensitivity of this technique in capturing subtle difference in T2 relaxation time between ischemic and remote myocardium [14, 17].

In Tertile-1, we found that patients with residual blood flow within AAR had lower infarct T2 values as compared to those without. Leftover blood flow in the territory at risk via either the infarct-related artery or collaterals is effective in protecting the myocardium from ongoing necrosis [22, 23]. Accordingly, recent evidences suggested that the severity of edema, as quantified by the lengthening of infarct T2 values, may be influenced by infarct-limiting interventions [9, 24]. Thus, cardioprotective strategies may reduce infarct T2 values, and further dedicated studies are need to specifically tackle this controversial aspect.

We also found that IMH influenced the pattern of T2 within infarct so that patients with IMH had a constant augmentation of T2 relaxation time after reperfusion. Moreover, Tertile-1 patients with IMH tended to have higher infarct T2 values than patients without IMH (*P* = 0.082). This result is in line with experimental data reporting that after reperfusion the increase of T2 values and water content in the infarct region were associated with the severity of IMH [6–9]. Overall the evidence converges in supporting the concept that IMH is not necessarily associated with a decrease of infarct T2 values, likely because T2 relaxation time represents the equipoise between the destructive paramagnetic effects of deoxyhemoglobin (T2 decline) and the increase of tissue water content (T2 raise), [6–9].

Finally, we observed that LGE-based estimate of infarct size was independent of the timing of CMR after reperfusion. This finding diverges from some previous clinical studies, which reported a time dependency of LGE extent prompting an overestimation of infarct size when CMR was performed early after reperfusion [9–12]. However, most of these studies included a small number of patients, and the first CMR was performed at 24 h and then repeated 1 week or later not allowing to adequately tracking infarct healing. In contradistinction, we divided the study cohort according to the timing of CMR after reperfusion. The tertiles thus obtained included more than 50 subjects for each, and they were evenly balanced with respect to clinical factors influencing I/R damage [13]. This allowed superseding some of the previous study limitations and to investigate how LGE-derived infarct size was influenced by the timing of CMR throughout a broad interval spanning from 6 h to 14 days after reperfusion. Furthermore, we measured ECV of the infarct region in patients without MVO, and this parameter remained stable across T_revasc-CMR_ tertiles, providing a mechanistic insight about the independency of LGE-derived infarct size from the timing of CMR.

This study has several limitations. Firstly, it was conducted in a tertiary referral center for PPCI and CMR. Accordingly, we cannot exclude a referral bias. T_revasc-CMR_ tertiles showed an even distribution of clinical and hemodynamic factors influencing I/R damage [11]. However, our study design cannot completely remove bias in allocating subjects to the 3 tertiles. Ideally, this limitation can be overcome by repeating CMR at fixed time points in large cohort of patients. However, this type of study entails complex protocols arising also ethical concerns due to repeated administration of gadolinium-based contrast agent for IS. To the best of our knowledge the largest study serially repeating contrast-enhanced CMR in the early post-STEMI included only 30 patients [5]. We did not use T2*-mapping for IMH identification and quantification to obviate to off-resonance artifacts which are particularly cumbersome at interface between myocardium and lung [25].

## Conclusion

In this study including consecutive unselected reperfused STEMI patients, CMR-derived parameters of I/R damage were not influenced by the timing of CMR after reperfusion. Although we acknowledge dynamic changes of T2 relaxation time of the infarct and the remote myocardium after revascularization, however these variations did not influence T2-mapping-derived AAR or LGE-based infarct size. Accordingly, CMR remains a valid non-invasive imaging modality for characterizing and quantifying the diverse components of I/R damage in STEMI patients during the early post-infarction phase.

## Abbreviations

AAR: Area at risk
bSSFP: Balanced steady state free precession
CMR: Cardiovascular magnetic resonance
ECG: Electrocardiogram
ECV: Extracellular volume
I/R: Ischemia/reperfusion
IMH: Intramyocardial hemorrhage
LGE: Late gadolinium enhancement
LV: Left ventricle/left ventricular
MOLLI: Modified Look-Locker inversion recovery
MVO: Microvascular obstruction
PPCI: Primary percutaneous coronary intervention
STEMI: St-segment elevation myocardial infarction
TIMI: Thrombolysis in myocardial infarction
